# Mechanical Dentistry

**Published:** 1875-11

**Authors:** 


### MECHANICAL DENTISTRY.

BY DR. CHISHOLM.

*Read before the Tennessee Dental Association.*

Mr. President and gentlemen, I am well aware of my inability to present you anything new in mechanical dentistry, not having been in regular practice since 1870. Nor do I expect to interest you but little, if at all, by a rehearsal of what all of you are more or less acquainted with. But the object of this paper is to add a mite, if possible, to the interest of the meeting by a mere statement of my limited experience and observations in this department of our calling.

The caption of this paper embraces a much larger field of thought and investigation than we propose to traverse, even were we able to do so. But as I have had but little or no experience in many of the materials used for dental bases, having confined my labors chiefly to gold and rubber, I propose to give only my little experience and observation in the use of these two materials.

Owing to the expense in both skill and material, the use of gold has almost become obsolete in this day of rubber rage, so much so indeed that many otherwise good operators know but little about its proper manipulation. Therefore to enter into minute details about this style of work, would most likely prove uninteresting on this occasion, as well as consume valuable time.

We will then speak only of some of the relative merits and demerits of this material compared with rubber, and pass on to a more extended consideration of the latter.

Gold, we claim, possesses some very desirable qualities for a base for teeth which are not found in rubber, among these is its susceptibility of thermal changes so pleasant in many instances to the patient, being a good conductor when anything cold or warm is taken in the mouth, the change is felt almost as readily as if there were no plate in it. This gives much pleasure to the patient in warm weather especialy. It is without doubt also, less objectionable to the mucous membrane of the mouth, whether this is due to the purity of the metal or the prompt changes of heat or cold, or both, I do not pretend to say, I have only observed it as a fact and leave you gentlemen to seek the cause.

I have thought that the articulation is better with a gold plate, in this, however, I may not be correct.

The surface of a gold plate is more easily cleaned than rubber.

We then sum up the advantages of gold over rubber as follows, viz:

1st. Facility of thermal changes.
2d. Health to gums.
3d. Favorable to articulation.
4th. Surface of plate easily cleansed.

The *disadvantages* are:

1st. Difficult manipulation.
2d. Weight.
3d. Difficult to cleanse as an apparatus.

This latter is the chief objection. The difficulty of fitting the teeth accurately to the plates leave, so many receptacles for foreign matter that the apparatus becomes offensive. It should be usually placed in a glass of water during the night as a kind of cleansing process. This fact, to me, somewhat accounts for the favorable condition of the gums with gold plate wearers.

I will now speak of some of the favorable qualities of rubber and my experience in its manipulation.

Rubber has become almost universally popular as a base for teeth. This arises, doubtless, from the ease with which it is worked, its easy adaptation to the parts, its cheapness, its strength and durability, and the ease with which it is cleansed as an apparatus. It possesses all these qualities over gold, and I leave you gentlemen to judge of the relative magnitude of these qualities between the two. Some objections in both styles of work could be avoided by uniting the two, this we have adopted of late in gold work and to our very great satisfaction, and hope the day is not distant when every upper plate will be gold with rubber attachments, as in this combination there would be one objectionable feature in both styles removed and one favorable one in both retained, cleanliness of the rubber as an apparatus, and thermal changes of the gold; both desirable, while the difficulty of manipulating the gold is greatly modified also.

We now propose to give our observations in the use of rubber.

One object to be attained in a denture, is *perfect adaptation*, and here I must be allowed to object to the phrase "*suction* plate." Atmospheric presure would be better, suction gives a wrong idea. We need only adaptation, suction only ends in this, and comfort begins where suction ends.

Why not avoid this and give comfort at once.

Our investigations in this direction, in 1866, induced us to abandon air chambers, and we assumed this possition in 1867 in a meeting at Memphis. Up to that time our mechanical labors were chiefly in gold work, and our success led us to feel quite master of the situation. But when rubber became fashionable, we confess we found ourselves in no little perplexity at times in the use of this material, to make that perfect adaptation so essential to comfort and success. But close attention to the difficulties led me, to some extent, to ascertain the causes, since which time I have had but little trouble and now I give my observations and remedy.

I have noticed that all the different preparations of rubber shrink more or less, and that shrinkage is in proportion to the extent it is vulcanized.

For instance, the Boston Star shrinks less than any I have tried, and if vulcanized a little green it shrinks less than when vulcanized hard, and so by all the other rubbers, only they shrink more. It is this shrinkage that must be overcome to make a success. Again, the shrinkage so detrimental to success is governed in a great degree by the curvatures in the plate. If the palatine arch is very deep the change will be greater than when shallow, yet if from the front to the back part of the plate be convex, which is usual, less damage is done than if the palatine arch were shallow with a concave surface from front backwards. We then set it down as a rule that in whatever direction a plate may curve, the shrinkage of the rubber increases that curvature and consequently two very important things are to be considered: First, the amount of shrinkage common to the rubber used, and second, the character of the curves in the plate to be made.

To make the proper allowance for these, constitutes the chief difficulty in close adaptation. Lower plates especially often rock and are quite unsteady from a want of attention to these two things, owing to their long curves in two directions.

To give specific directions how to remedy these difficulties is more than I would at present attempt to do, suffice it to say, that experience is better than twenty masters.

We always take impressions in plaster if possible, on this we make all the changes that experience suggests by a piece of rolled paper, camel's hair pencil and some times a delicate scraper, but such things must be used with great care and caution, after the proper allowance is made in the impression for the usual shrinkage of the rubber, the models are gotten up and the work done in the usual way. Sometimes however, when the desired change can not be made in the impression, I make it on the modle.

In taking occlusion bites I use a small stiff wire, placed on the wax plate at the time to support it firmly and avoid its getting out of position at the time, this is very essential in lower plates.

In antagonising full sets it is best to have the grinding surfaces of the molars on a plane than to be locked into each other, as a slight latteral or forward move of the jaw would be inclined to disturb their position.

